# Awareness and Knowledge of Venous Thromboembolism Among Saudi Adults in the Dawadmi Province: A Cross-Sectional Study

**DOI:** 10.7759/cureus.52742

**Published:** 2024-01-22

**Authors:** Essam Elmahdi, Badreldin A Yousif, Mohammed Nawar Alotaibi, Mohammad A Rashikh, Nemer Alotaibi, Saad Alsaab, Abdulmgeed F Alruways, Abdulaziz Aladhyani, Mohannad M Aljuaid, Eid H Alotaibi, Majed R Alharthy, Hallal Alotaibi, Dayis S Alqahtani

**Affiliations:** 1 Internal Medicine, College of Medicine, Shaqra University, Dawadmi, SAU; 2 Internal Medicine, Dawadmi General Hospital, Dawadmi, SAU; 3 Medicine, College of Medicine, Shaqra University, Dawadmi, SAU; 4 Pharmacology and Therapeutics, College of Medicine, Shaqra University, Dawadmi, SAU; 5 Pediatrics, College of Medicine, Shaqra University, Dawadmi, SAU; 6 Family and Community Medicine, College of Medicine, Shaqra University, Dawadmi, SAU; 7 Medicine, Dawadmi General Hospital, Dawadmi, SAU

**Keywords:** kingdom of saudi arabia (ksa), obesity, cigarette smoking, pulmonary embolism, deep vein thrombosis, immobility, knowledge level

## Abstract

Background

Venous thromboembolism (VTE) significantly contributes to the global disease burden. The annual incidence of VTE is one to two per 1,000 adults worldwide. We aimed to evaluate the awareness and knowledge of VTE risk factors, manifestations, prevention, and treatment options among the general adult population of Dawadmi, Riyadh, Saudi Arabia.

Methodology

We conducted a cross-sectional study using the self-designed and validated VTE knowledge questionnaire. A survey was conducted online via Google Documents, composed of 12 questions. Participants included in the study were over 18 years old, regardless of their medical history. However, medical students and healthcare providers were excluded.

Results

A total of 384 participants (46.4% men and 53.6% women) completed the survey; most respondents were between 18 and 28 years of age. Majority of the participants recognized that immobility, obesity, complicated surgery, road traffic accidents, smoking, and old age are the most common risk factors for VTE. However, more than half of the elderly and lower-educated participants did not identify complicated surgery and consumption of oral contraceptive pills as risk factors for VTE. Awareness of VTE was significantly higher among those with a family history of VTE and a graduate degree (p<0.001). Results showed a deficit of awareness and information about VTE, especially among males, those with low education, and elderly participants.

Conclusion

This study demonstrates the need for more awareness of VTE among the Saudi adult population. The urge to spread awareness and knowledge about VTE among the public in Dawadmi province is required.

## Introduction

Venous thromboembolism (VTE, i.e., deep vein thrombosis [DVT] and pulmonary embolism [PE]) is a prominent contributor to the global disease burden [1]. It affects thousands of patients worldwide and is a cause of annual hospitalizations [2]. The annual incidence of VTE is one to two per 1,000 adults worldwide [1,3]. Several risk factors are associated with thrombosis, including older age, obesity, smoking, history of thrombosis, surgery, hospitalization, varicose veins, thrombophilia, oral contraceptives, and pregnancy [4-7]. Worldwide, PE, especially in postpartum and pregnant women, is the cause of maternal death [8]. Hospital-associated VTE is a leading cause of mortality and disability [1]. However, VTE-related mortality and disability are mainly preventable [9]. Thromboprophylaxis among risky individuals can minimize morbidity and mortality [10-12]. To upsurge global awareness of the thrombosis-related disease burden, starting in 2014, the International Society on Thrombosis and Haemostasis (ISTH) declared 13 October as the World Thrombosis Day [13]. A few studies in the Kingdom of Saudi Arabia have shed light on community awareness of VTE [2,3,14]; hence, people must be aware of this disorder as it may be a life-threatening disease [13]. This study aimed to assess the awareness and knowledge of VTE risk factors, manifestations, prevention, and treatment options among the general adult population of Dawadmi, Riyadh, Saudi Arabia.

## Materials and methods

Study design

We conducted a cross-sectional study and distributed a survey questionnaire to the adult population of Dawadmi between March 2022 and July 2022. Participants were excluded if they were working or studying in the medical field.

Sample size

The sample size was calculated using OpenEpi software and the following formula:

n = Z2∗P(1 − P)/e2

where n is the sample size, Z is the level of confidence (95% confidence interval =1.96), P is the prevalence of VTE (1-2 per 1000 adults worldwide) [1,3], and e is the margin of error.

The minimum recommended sample size for this study was 384.

The inclusion criteria were age ≥18 years, Saudi by citizenship, the general adult population of Dawadmi city, and both genders. The exclusion criteria were age <18 years, medical students, and healthcare providers.

Survey instrument

After the ethical committee approved the study, we conducted a cross-sectional study using an online questionnaire survey of 12 questions distributed through social media and hard copies in the Dawadmi province. The questionnaire was developed by combining two previously validated surveys [1,15]. The questionnaire was translated into Arabic and validated in a pilot study involving 20 Arabic-speaking participants. The questionnaire survey consisted of demographic information including age, sex, level of education, personal or family history of VTE, awareness of DVT and PE, including their underlying risk factors, signs and symptoms, and prevention. Consent was obtained from each participant before data collection. At the end of the questionnaire, an email was provided to the participants to inquire about any unclear questions. The completed questionnaires were collected and kept with the principal investigators. Data were strictly protected for confidentiality when conducting the study.

Statistics

Data entry and analysis were conducted using SPSS Version 25 (IBM Corp. Armonk, NY). Categorical variables were presented as frequency and percentage. The Chi-square (χ2) test of independence was applied to examine the statistically significant association between two categorical variables. All comparisons were considered significant at p<0.05.

Ethical approval

The study obtained ethical approval from the Institutional Ethics Committee at Shaqra University (approval number: ERC_SU_24220075) and the local Research Ethics Committee, College of Medicine, Shaqra University, Dawadmi (project number: CMD/DWD/SU/2022/04/067).

## Results

Of the 440 screened participants, 384 completed the questionnaire, constituting an 87.3% response rate. As shown in Table 1, 53.4% of respondents were below 28 years of age, and 53.6% were female. Most respondents had a bachelor's degree (52.1%). Only 21.4% of respondents had a family history of VTE.

**Table 1:** Demographic and personal information of participants (N = 384) N, total number of participants; VTE, venous thromboembolism

Characteristics		Frequency (n)	Percentage (%)
Gender of parents	Male	178	46.4
	Female	206	53.6
Age groups (in years)	18-28	205	53.4
	29-38	81	21.1
	39-48	70	18.2
	49-58	22	5.7
	59-68	5	1.3
	69-78	1	0.3
Educational level	Primary school	11	2.9
	Middle school	36	9.4
	High school	131	34.1
	Bachelor	200	52.1
	Illiteracy	6	1.6
Family history of VTE	Yes	82	21.4
	No	302	78.6

**Figure 1:**
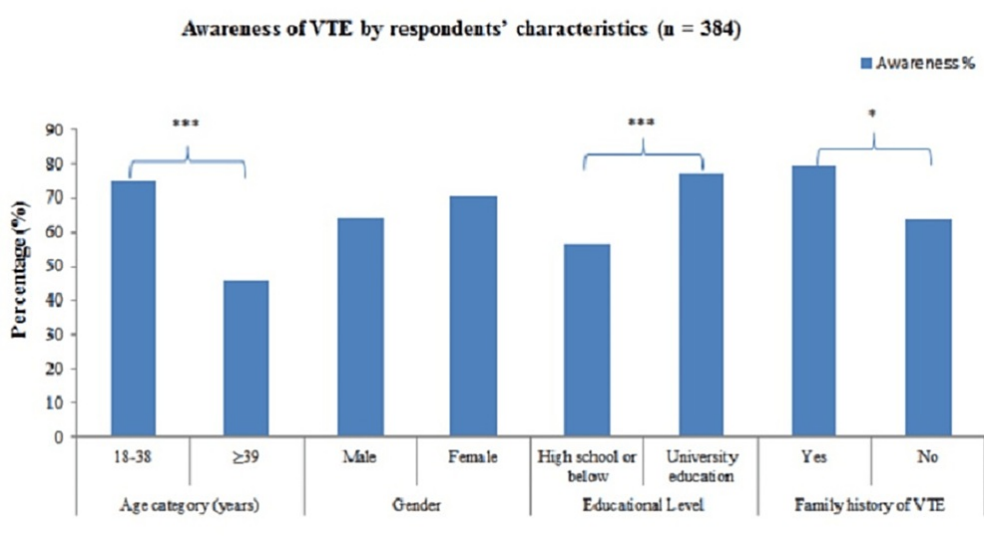
Respondents’ awareness of VTE VTE, venous thromboembolism; n, number of participants *Significant; ***Highly significant

Figure 1 shows the respondents’ awareness of VTE by age, sex, level of education, and personal and family history of VTE. The percentage of respondents reporting awareness of VTE was significantly higher among those with higher education (77.5% versus 56.5%; p<0.001) and a personal or family history of VTE (79.3% versus 63.9%; p<0.05). Moreover, awareness of VTE was significantly associated with educational level, family history of VTE, and age of participants (p<0.05), but it was insignificant with gender (p>0.05).

As shown in Table 2, women had a better understanding of the severe consequences of VTE than men, as 72.3% of women could understand its danger compared to 53.4% of the men group (p<0.001). Similarly, regarding the manifestations of DVT among participants, women had good knowledge compared to men (68.5% vs 39.5%; p<0.001). Regarding medicines used in cases of VTE, we found that women had a better understanding than men, as 68% of women knew that anti-coagulation was the correct answer, which matched 53.9% of the men group (p<0.05). Furthermore, females had a better understanding of preventive measures than males (p<0.05).

**Table 2:** Participants' knowledge and awareness about the risk factors, symptoms, prevention, and medication used in VTE, as categorized by gender (n =384) n, frequency; VTE, venous thromboembolism; PE, pulmonary embolism; DVT, deep vein thromboembolism ^a^Based on the chi-square test, *Significant

Characteristics		Male, n (%)	Female, n (%)	p-Value^a^
		Total n %, 178 (46.4)	Total n %, 206 (53.6)	
Are you diagnosed with VTE or have a family history of VTE?		37 (20.8)	45 (21.8)	0.801
Do you consider VTE/DVT dangerous and can lead to PE?		95 (53.4)	149 (72.3)	0.000*
Knowledge about the risk factors of VTE/DVT	Sedentary lifestyle	142 (79.8)	177 (85.9)	0.109
	Complicated surgery	85 (47.8)	125 (60.7)	0.011*
	Smoking	105 (59)	117 (56.8)	0.664
	Obesity	107 (60.1)	128 (62.1)	0.685
	Road traffic accident	127 (71.3)	135 (65.5)	0.222
	Family history of VTE	90 (50.6)	102 (49.5)	0.838
	Age more than 60 years	104 (58.4)	136 (66)	0.125
	Use of oral contraceptive pills	75 (42.1)	115 (55.8)	0.007*
Manifestations of VTE/DVT	Pain in the leg	70 (39.3)	30 (14.6)	0.000*
	Redness and swelling of the legs	9 (5.1)	8 (3.9)	
	Redness or color change	12 (6.7)	8 (3.9)	
	Swelling	8 (4.5)	10 (4.9)	
	All of the above	71 (39.9)	141 (68.5)	
Prevention of VTE/DVT	Walking and sitting for a long time	46 (25.8)	32 (15.5)	0.005*
	Exercise	18 (10.1)	13 (6.3)	
	Medications to high-risk patients	9 (5.1)	13 (6.3)	
	Weight reduction	10 (5.6)	4 (1.9)	
	All of the above	95 (53.4)	144 (69.9)	
Medication use in VTE/DVT	Anti-coagulants	96 (53.9)	140 (68)	0.015*
	Anti-histamines	47 (26.4)	34 (16.5)	
	Diuretics	35 (19.7)	32 (15.5)	

As shown in Table 3, younger participants had a significantly higher knowledge about VTE consequences, manifestation, and prevention of DVT than the older age group (p<0.001). On the contrary, an insignificant relationship was observed between VTE medication and participants' age group (p>0.05). Moreover, most study participants recognized the risk factors of VTE occurrence. They found a significant relationship between specific risk factors of VTE (such as the use of contraceptive pills, surgery, and people older than 60 years) and age group (p<0.05).

**Table 3:** Participants’ knowledge and awareness about the risk factors, symptoms, prevention, and medication used in VTE, as categorized by different age groups n, frequency; VTE, venous thromboembolism; DVT, deep vein thromboembolism; PE, pulmonary embolism ^a^Based on the chi-square test, *Significant

Characteristics		Younger age group (≤38 years), n (%)	Older age group (≥39 years), n (%)	p-Value^a^
		Total n (%), 286 (74.47)	Total n (%), 98 (25.5)	
Are you diagnosed with VTE or have a family history of VTE?		67 (23.4)	15 (15.3)	0.090
Do you consider VTE/DVT dangerous and can lead to PE?		207 (72.4)	37 (37.8)	0.000*
Knowledge about the risk factors of VTE/DVT	Sedentary lifestyle	243 (85.0)	76 (77.6)	0.091
	Complicated surgery	167 (58.4)	43 (43.9)	0.013*
	Smoking	166 (58.0)	56 (57.1)	0.876
	Obesity	171 (59.8)	64 (65.3)	0.334
	Road traffic accident	199 (69.6)	63 (64.3)	0.331
	Family history of VTE	143 (50.0)	49 (50.0)	1.000
	Age more than 60 years	189 (66.1)	51 (52.0)	0.013*
	Use of oral contraceptive pills	152 (53.1)	38 (38.8)	0.014*
Manifestation of VTE/DVT	Pain in the leg	66 (23.1)	34 (34.7)	0.000*
	No symptoms	10 (3.5)	7 (10.2)	
	Redness and swelling of the legs	7 (2.4)	10 (10.2)	
	Redness or color change	13 (4.5)	7 (7.1)	
	Swelling	13 (4.5)	18 (4.7)	
	All of the above	177 (61.9)	35 (35.7)	
Prevention of VTE	Walking and sitting for a long time	50 (17.5)	28 (28.6)	0.000*
	Exercise	23 (8)	8 (8.2)	
	Medications to high-risk patients	14 (4.9)	8 (8.2)	
	Weight reduction	4 (1.4)	10 (10.2)	
	All of the above	195 (68.2)	44 (44.9)	
Medication used in VTE	Anti-coagulants	181 (63.3)	55 (56.1)	0.293
	Anti-histamines	55 (19.2)	26 (26.5)	
	Diuretics	50 (17.5)	17 (17.3)	

As shown in Table 4, higher-educated respondents had better knowledge and awareness about DVT risk factors, consequences, manifestation, prevention, and medication used in VTE than lower-educated respondents (p<0.001). Moreover, a significant relationship was observed between knowledge about the risk factors, consequences, manifestation, prevention, and medication used in VTE and the educational level of participants (p<0.001).

**Table 4:** Participants’ knowledge and awareness about risk factors, symptoms, prevention, and medication used in VTE, as categorized by level of education n, frequency; VTE, venous thromboembolism; DVT, deep vein thromboembolism; PE, pulmonary embolism ^a^Based on the chi-square test, *Significant

Characteristics		Lower education, n (%)	Higher education, n (%)	p-Value^a^
		Total n (%), 184 (47.9)	Total n (%), 200 (52.1)	
Are you diagnosed with VTE or have a family history of VTE?		37 (20.1)	45 (22.5)	0.568
Do you consider VTE/DVT dangerous and can lead to PE?		86 (46.7)	158 (79.0)	0.000*
Knowledge about the risk factors of VTE/DVT	Sedentary lifestyle	146 (79.3)	173 (86.5)	0.062
	Complicated surgery	87 (47.3)	123 (61.5)	0.005*
	Smoking	97 (52.7)	125 (62.5)	0.052
	Obesity	98 (53.3)	137 (68.5)	0.002*
	Road traffic accident	126 (68.5)	136 (68.0)	0.920
	Family history of VTE	75 (40.8)	117 (58.5)	0.001*
	Age more than 60 years	100 (54.3)	140 (70.0)	0.002*
	Use of oral contraceptive pills	71 (38.6)	119 (59.5)	0.000*
Manifestations of VTE/DVT	Pain in the leg	64 (34.8)	36 (18)	0.000*
	No symptoms	13 (7.1)	4 (2)	
	Redness and swelling of the legs	12 (6.5)	5 (2.5)	
	Redness or color change	12 (6.5)	8 (4)	
	Swelling	12 (6.5)	6 (3)	
	All of the above	71 (38.6)	141 (70.5)	
Prevention of VTE/DVT	Walking and sitting for a long time	50 (27.2)	28 (14)	0.000*
	Exercise	20 (10.9)	11 (5.5)	
	Medications to high-risk patients	13 (7.1)	9 (4.5)	
	Weight reduction	13 (7.1)	1 (0.5)	
	All of the above	88 (47.8)	151 (75.5)	
Medication use in VTE/DVT	Anti-coagulants	94 (51.1)	142 (71)	0.000*
	Anti-histamines	58 (31.5)	23 (11.5)	
	Diuretics	32 (16.4)	35 (17.5)	

## Discussion

Thromboprophylaxis in hospitalized patients decreases the incidence of VTE significantly; however, the lack of public awareness and knowledge of VTE and management put the population at higher risk, which leads to morbidity and mortality [16]. Thus, multiple calls exist to enhance public awareness of VTE [17]. The current study was designed to evaluate the extent of the public knowledge and perception of the risk factors, symptoms, signs, and prevention of VTE.

In general, we found that most participants had good knowledge of VTE; two-thirds knew that VTE is a condition with high mortality, and 90% knew of the different signs and symptoms. The results of the present study are inconsistent with some previous studies, including the study of Okoye et al., who found that there are generally low levels of awareness of VTE as a medical condition [15]. A previous global study found a need for more public awareness concerning thrombotic disorders and VTE [1]. Another study found that there is significant poverty awareness about VTE [18], and this low awareness was reported in several studies in the outpatient setting [19], among medical students [20], hospitalized patients [21], and hospital staff [22]. Moreover, several studies conducted in different developed countries have shown a decreased awareness among the general population regarding VTE, including a study conducted in the United States of America [23] and Australia [24].

Furthermore, another cross-sectional study conducted in Jordan to evaluate the level of awareness of patients who had cesarean sections concerning VTE found that there was a lack of awareness of VTE among patients in general and among the young participants in particular, where 46% and 18.7% were aware of DVT and PE, respectively [25]. Another study conducted by Sousou and Khorana on ambulatory active cancer patients showed that more than half of the participants were unaware of the increased risk of VTE with cancer [26]. The higher level of awareness and knowledge regarding VTE in our survey compared to other studies may be related to the high education level and young age of the public enrolled in the current study.

Concerning the participants’ knowledge about the risk factors associated with VTE, we found that most participants were aware of the risk factors and incidence of VTE. A previous study found that participants need to have more knowledge regarding VTE’s risk factors and preventive strategies, which are necessary for hospitalized patients to actively participate in preventing VTE [27]. Moreover, other studies showed that some risk factors, including surgeries, pregnancy, cancer, and family history, were not reported by the participants in their studies [1,15]. In addition, another study showed that most participants who identified VTE risk factors correctly recognized immobility as the leading risk factor for DVT and PE [21].

Our study found that most participants had good knowledge concerning different strategies for preventing VTE. Also, we found that most participants were aware of the impact of oral contraceptive pills and other medications that could account for VTE. In a study by Okoye et al., the authors reported a low level of awareness by the general population regarding the risk factors and manifestations of VTE [15].

In the present study, we assessed the factors that affect the knowledge and awareness of the participants in the matter of VTE. The age of the participants was a significant factor that affected the degree of familiarity regarding VTE, whereas older participants significantly had a higher level of knowledge. Furthermore, the educational level positively impacted the overall awareness since the higher the level of education, the better the understanding of the risk factors of VTE. A previous study conducted by Oh et al. showed that the awareness of the manifestations of stroke was higher among educated individuals and those who got informed through campaigns, public education programs, and websites [27]. A previous randomized controlled study showed that the level of knowledge and awareness about VTE was significantly higher after the educational programs introduced by the nurses toward postpartum women and developed from 8% to 87% [28]. This result showed that encouraging patients and public individuals to be involved in educational programs toward VTE, its manifestation, risk factors, and prevention will decrease the incidence of hospitalized acquired VTE.

Considering the difference in response according to the gender of the participants, the results of this study showed a significant difference between the two groups concerning their knowledge of VTE. Females have a significantly higher awareness of VTE’s predisposing factors and preventive strategies; this might be attributed to the fact that some women in the study had already experienced an episode of VTE. Also, generally, women tend to show more interest in public awareness. Our study results are consistent with a previous study, which showed that females and individuals below 35 were more likely to be better informed than males about VTE symptoms and risk factors [15].

Based on our results, we recommended increasing educational programs on VTE to encourage active involvement of patients in treatment plans, ensure their adherence, promote self-diagnosis and reporting of VTE symptoms, and reduce the incidence of VTE among the general public. Educational campaigns can be beneficial and effectively increase public awareness about VTE [29].

Study limitations

The limitations of this study include depending on a self-reported questionnaire, which could lead to personal bias, where some participants may choose answers randomly. Moreover, depending on the online means for the questionnaire’s distribution could lead to sampling bias toward younger and educated populations more familiar with VTE.

## Conclusions

In conclusion, we found a good level of awareness among the general population regarding VTE, symptoms, risk factors, and prevention strategies. However, most participants did not identify complicated surgery and use of oral contraceptive pills as risk factors for VTE, especially among males, lower educated individuals, and the elderly. An education campaign should be conducted to maximize public awareness of VTE. Further studies involving other regions are warranted.
